# Effect of administration routes of oxytocin on hemoglobin in neonates with delayed umbilical cord clamping: a multi-centre randomized controlled clinical trial

**DOI:** 10.1007/s00404-024-07543-w

**Published:** 2024-05-16

**Authors:** Lu Mei, Ning Gu, Yan Zhou, Zhiqun Wang, Ling Yang, Li Chen, Chunxia Li, Yimin Dai

**Affiliations:** 1 Department of Obstetrics and Gynaecology, Nanjing Drum Tower Hospital, Nanjing Drum Tower Hospital Clinical College of Nanjing Medical University, Nanjing, 210008 China; 2 Department of Obstetrics and Gynaecology, Nanjing Jiangning Hospital of Chinese Medicine, Nanjing, 211100 China; 3 Department of Neonatology, Nanjing Drum Tower Hospital, Nanjing Drum Tower Hospital Clinical College of Nanjing Medical University, Nanjing, 210008 China; 4 Department of Obstetrics and Gynaecology, Yangzhong People’s Hospital, Yangzhong, 212200 China

**Keywords:** Delayed cord clamping, Oxytocin, Cesarean section, Intramyometrial injection, Intravenous infusion, Neonatal hemoglobin

## Abstract

**Purpose:**

To evaluate the effect of intravenous infusion versus intramyometrial injection of oxytocin on hemoglobin levels in neonates with delayed umbilical cord clamping during cesarean section.

**Methods:**

The multi-centre randomized controlled trial was performed at three hospitals from February to June 2023. Women with term singleton gestations scheduled for cesarean delivery were allocated to receive an intravenous infusion of 10 units of oxytocin or a myometrial injection of 10 units of oxytocin during the surgery. The primary outcome was neonatal hemoglobin at 48 to 96 h after birth. Secondary outcomes were side-effects of oxytocin, postpartum haemorrhage, phototherapy for jaundice, feeding at 1 month, maternal and neonatal morbidity and re-admissions.

**Results:**

A total of 360 women were randomized (180 women in each group). The mean neonatal hemoglobin did not show a significant difference between the intravenous infusion group (194.3 ± 21.7 g/L) and the intramyometrial groups (195.2 ± 24.3 g/L) (*p* = 0.715). Secondary neonatal outcomes, involving phototherapy for jaundice, feeding at 1 month and neonatal intensive care unit admission were similar between the two groups. The maternal outcomes did not differ significantly between the two groups, except for a 200 mL higher intraoperative infusion volume observed in the intravenous group compared to the intramyometrial group.

**Conclusion:**

Among women undergoing elective cesarean delivery of term singleton pregnancies, there was no significant difference in neonatal hemoglobin at 48 to 96 h after birth between infants with delayed cord clamping, whether the oxytocin was administrated by intravenous infusion or intramyometrial injection.

**Trial registration:**

Chinese Clinical trial registry: ChiCTR2300067953 (1 February 2023).

**Electronic supplementary material:**

The online version of this article (10.1007/s00404-024-07543-w) contains supplementary material, which is available to authorized users.

## What does this study adds to the clinical work

**Table:** 

Different administration route for oxytocin during elective cesarean delivery does not significantly affect its effectiveness for women undergoing DCC with term singleton gestations.	

## Introduction

Delayed cord clamping (DCC) is the practice of clamping the umbilical cord at least 60 s after birth or after the cessation of umbilical cord pulsation (usually performed within 1–3 min after birth) [1]. In comparison to immediate cord clamping, DCC promotes continuous blood flow between the placenta and the newborn during the third stage of labor, ensuring a smooth hemodynamic transition for the infant [2]. It has been shown to enhance neonatal hemoglobin and decrease the risk of neonatal anemia [3–5], in addition to improving iron storage and long-term neurodevelopmental outcomes [6, 7]. Moreover, DCC does not contribute to maternal blood loss, postpartum hemorrhage incidence or the need for phototherapy for jaundice [8, 9].

The factors affecting placental transfusion during DCC include the frequency and intensity of uterine contractions, the duration of uterine contractions, the position of the newborn, the timing of the neonate’s spontaneous breathing, and the method employed for DCC. Notably, uterine contraction is the main determinant [10]. Almost all of the placental transfusion volume can be obtained within one minute if the uterotonic agent is administered intravenously during delayed umbilical cord clamping [11]. Consequently, most major international guidelines recommend that the duration of DCC should be one minute [12, 13]. Given the absence of antepartum uterine contractions in elective cesarean sections, the timing and intensity of postpartum spontaneous contractions, as well as the type and administration route of uterotonic agents, could potentially influence the uterine contractility within the first minute postpartum, which impact the volume of placental transfusion. However, there is a lack of research investigating the effects of uterotonics which are commonly employed by current guidelines on DCC during cesarean section.

Oxytocin is the first-line agent for the management of postpartum hemorrhage (PPH) [14]. The administration of oxytocin after delivery is recommended by the World Health Organization (WHO) as a part of the active management of the third stage of labor. It can be administered intravenously and/or intramuscularly [15]. Nevertheless, no priority was recommended, primarily because there was almost no difference between the two administration routes in terms of the incidence of postpartum hemorrhage or the occurrence of side-effects [16]. Intramyometrial injection of oxytocin is a common practice in China and Japan [17], and is also used in other countries such as Canada and India [18]. In clinical practice, some hospitals choose intramyometrial injection alone to reduce side-effects associated with intravenous route of the uterotonic agents, while others prefer intravenous infusion to avoid injecting injuries during surgery. A considerable number of hospitals are now adopting simultaneous intramyometrial injection and intravenous injection of oxytocin to better prevent PPH, but there is no emphasis on prioritizing these methods. According to previous studies, intravenous oxytocin has an immediate effect, while intramuscular uterine injection has an effect within 3 to 7 min [19]. The intensity of uterine contraction induced by intravenous administration is greater than that induced by myometrial injection within the first minute [16, 20]. Therefore, we hypothesized that placental transfusion would be more effective, when oxytocin was administrated immediately through intravenous infusion rather than intramyometrial injection following fetal delivery.

In this multicenter randomized controlled clinical trial, our objective was to compare the effects of intravenous infusion with intramyometrial injection of oxytocin during cesarean section on neonatal hemoglobin after DCC, aiming to provide clinical evidence for selecting the preferred route of oxytocin administration during cesarean section.

## Methods

### Study design

This multi-centre, open-label, parallel, randomized controlled clinical trial was conducted at three hospitals in East China, between February 2023 and June 2023.The three hospitals, a tertiary referral hospital, a tertiary Chinese medicine hospital and a secondary general hospital, have approximately 10,000 deliveries annually. The study protocol was approved by the relevant ethics committee and regulatory agencies at each site (available in the supplementary material). No interim analysis was planned. Prior to starting the trial, all the staff were trained according to the standard operative procedure, and written informed consent was obtained from all participants. Surgeries were performed by experienced or supervised obstetricians. This study was registered with the Chinese Clinical Trial Registry (ChiCTR2300067953, http://www.chictr.org.cn) and the CONSORT guidelines were followed.

### Study population

Women planned for cesarean delivery at the participating hospitals were screened for eligibility on a daily basis. The inclusion criteria were as follows: (1) maternal age ≥ 18 years old (2) gestational age ≥ 37 weeks (3) singleton gestation (4) elective lower segment cesarean delivery under spinal anesthesia. The exclusion criteria were: (1) women with placenta previa or placenta abruption (2) prenatal diagnosed fetal anomalies (3) suspected fetal anemia (4) fetal growth restriction with abnormal Doppler (5) multiple gestations (6) severe maternal cardiovascular disease (7) preoperative hemoglobin ≤ 70 g/L or (8) general anesthesia or allergies to oxytocin.

### Randomization

The randomization sequence was generated by a senior biostatistician via SAS software with a 1:1 allocation ratio into either the intramyometrial group or intravenous group. Randomization was stratified based on the study centers. The number of hospital births was as follows: 272 in Nanjing Drum Tower Hospital, 48 in Nanjing Jiangning Hospital of Chinese Medicine, and 40 in Yangzhong People’s Hospital. The sequence was placed into numbered opaque envelopes by an uninvolved third party before the initiation of the study. Eligible women were approached by obstetric clinicians on the day of the operation or the day before surgery. In the operating room, the obstetrician opens a random envelope according to the patient’s enrollment number and issues preoperative medical orders according to the group information in the envelope. Given the nature of the interventions, blinding was infeasible for of obstetricians, intraoperative research staff, and patients.

### Sample-size calculation

We conducted a preliminary experiment to calculate the sample size by collecting data from 11 patients from Nanjing Drum Tower Hospital. Among women who underwent elective cesarean section, the neonatal hemoglobin was 216.2 ± 12.6 g/L in the intravenous group and 211.2 ± 19.5 g/L in the intramyometrial group at 48–96 h of life. The sample size was calculated using PASS 15.0 software. Group sample sizes of 171 and 171 achieved 80.0% power to reject the null hypothesis of equal means when the population mean difference was 5.0 with standard deviations of 19.5 for the intramyometrial group and 12.6 for the intravenous group, and with a significance level (α) of 0.05 using a two-sided two-sample unequal-variance *t* test. The sample size increased by 5% to be 180 for each group to allow for dropout.

### Interventions

Cesarean section was performed in the supine position and Pfannenstiel incisions were used. Following delivery, the neonate was placed between the mother’s legs, and the umbilical cord was kept free of tension. One surgeon dried the newborn on the sterile field and covered the baby with dry cloth to keep it warm. For patients in the intravenous group, 10 IU of oxytocin was diluted in 500 mL of lactated Ringer’s solution by a nurse and administered through gravity-driven infusion with the roller clamp fully open intravenously. For women allocated to the intramyometrial group, oxytocin 10 IU (1 mL) of oxytocin was administered into the myometrium of the upper margin of the lower uterine segment incision immediately after the fetus was removed from the uterus by an assistant surgeon. At 60 s, the surgeons were called to clamp the umbilical cord. Early or immediate cord clamping (within 60 s after birth) should be performed when the newborn requires resuscitation with positive-pressure ventilation, or other conditions based on physician discretion.

After placenta delivery, the uterus incision was closed continuously in two layers. Additional management of blood loss was at the discretion of the obstetrician in line with routine practice at each hospital.

Neonates received routine care. Serum total bilirubin was measured when the transcutaneous bilirubin value exceeded the 75th percentile of the age-bilirubin curve [21]. All newborn blood samples were collected by the heel-stick method, which is the time of routine newborn metabolic screening in our country. If this sample could not be obtained, we did not perform an additional neonatal heel-stick, and the hemoglobin level was not measured. Telephone follow-up visits were conducted 42 days after delivery.

### Outcomes

The primary outcome was neonatal hemoglobin at 48 to 96 h after birth. Secondary neonatal outcomes were neonatal hematocrit at 48 to 96 h after birth, neonatal complications (hypothermia, anemia, infections, neonatal hypoglycemia), neonatal intensive care unit (NICU) admission, neonatal death, feeding at 1 month, re-admission within 30 days, and phototherapy for jaundice. The initiation of phototherapy occurred once the serum bilirubin level of the newborns reached the threshold for phototherapy [22]. Secondary maternal outcomes included estimated and calculated blood loss, additional uterotonics for excessive bleeding, surgical interventions, side-effects of oxytocin (hypotension, tachycardia), blood transfusion, ICU admission, and re-admission within 42 days. Postpartum hemorrhage was defined as an estimated blood loss greater than 1000 mL [23]. Neonatal anemia is defined as capillary vascular hemoglobin ≤ 145 g/L within 2 weeks of birth [24]. Neonatal hypothermia was defined as a neonatal axillary temperature less than 36.5 °C [25]. Neonatal hypoglycemia was defined as blood glucose < 2.6 mmol/L after 6 h postnatal [26]. The diagnostic criteria for ABO-related hemolytic disease in newborns included clinically significant hyperbilirubinemia, ABO incompatibility between mothers and neonates, and a positive direct antiglobulin test (direct Coombs test) on neonatal red cells [27].

### Statistical methods

Statistical analysis was performed with SPSS 25.0 software. The data were analyzed based on intent-to-treat. Normally distributed data are presented as the mean ± standard deviation (SD), and between-group differences were calculated using independent samples *t* tests. Skewed data are shown as the median and interquartile range (IQR) and differences between groups were analyzed using the Mann‒Whitney U test. Categorical variables are summarized as numbers and percentages (%), and between-group differences were analyzed using the Chi-square test or Fisher’s exact test as appropriate. All *P* values were two-sided, and values below 0.05 were considered statistically significant.

## Results

**Fig. 1:**
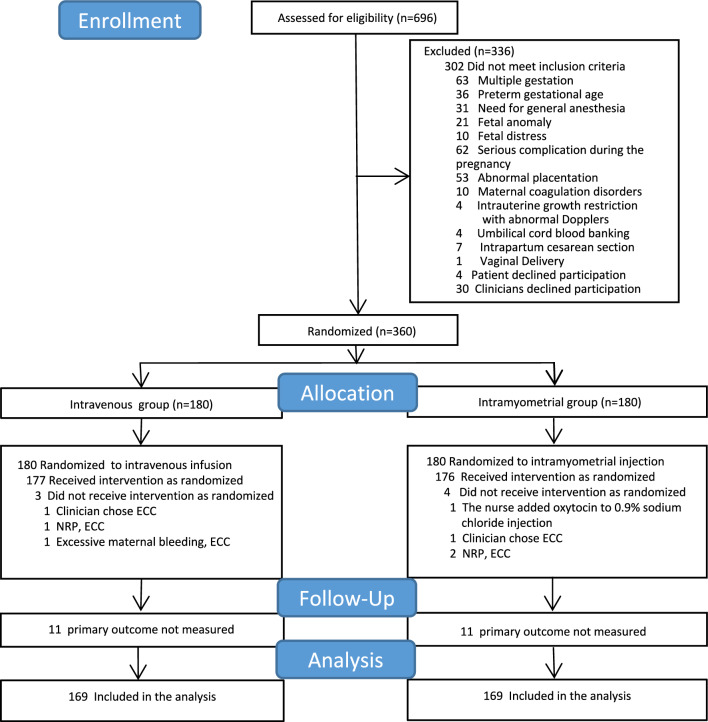
Study flow chart. *NRP* Neonatal Resuscitation Program; *ECC* Early Cord Clamping (within 60 s after birth)

Study enrollment began in February 2023 and was completed in June 2023. During this period, 696 women were screened for eligibility. A total of 360 women were enrolled and randomized (180 in the intravenous group and 180 in the intramyometrial group) (Fig. 1). Among them, 272 were from Nanjing Drum Tower Hospital, 48 were from Nanjing Jiangning Hospital of Chinese Medicine, and 40 were from Yangzhong People’s Hospital. The follow-up for the whole study was completed in August 2023. The data from all participants were analyzed based on their initial group allocation. We excluded 22 cases with missing primary outcome data before analysis. The baseline characteristics of the participants were comparable between the two groups (Table 1). The mean preoperative hemoglobin was similar between the two groups (122.7 g/L vs. 123.8 g/L, *p* = 0.335). All operations were performed under intraspinal anesthesia. The median time for umbilical cord clamping was 65 s in both the intravenous group (IQR, 62–68 s) and the intramyometrial group (IQR, 62–67 s), with no significant difference observed (*p* = 0.188). As is known that ABO hemolytic disease may affect hemoglobin level, we excluded six hemolytic samples before analyzing the neonatal hemoglobin and neonatal anemia.

**Table 1:** Baseline characteristics of the two groups

Characteristics	Intravenous group (*n* = 169)	Intramyometrial Group (*n* = 169)	*P* value
Maternal age, mean ± SD, years	31.6 ± 4.6	31.4 ± 4.4	0.678
Gestational age at delivery, median (IQR), week	39 (38.6, 39.2)	39 (38.6, 39.3)	0.415
Pre-delivery BMI, median (IQR), kg/m^2^	27.8 (25.7,30.0)	28.0 (26.1,30.7)	0.201
Primigravida, *n* (%)	95 (56.2)	90 (53.3)	0.585
Preoperative haemoglobin (g/L, mean ± SD)	122.7 ± 10.6	123.8 ± 10.4	0.335
Hemoglobin < 110 g/L, *n* (%)	15(8.9)	11 (6.5)	0.414
71–89	0	0	–
90–99	2 (1.2)	0	0.478
100–109	13 (7.7)	11 (6.5)	0.672
Hypertensive disorder, *n* (%)	9 (5.3)	10 (5.9)	0.813
Gestational diabetes, *n* (%)	32 (18.9)	24 (14.2)	0.242
Hepatitis B virus infection, *n* (%)	3 (1.8)	8 (4.7)	0.125
Main indications for cesarean section, *n* (%)			
Previous cesarean section	67 (39.6)	67 (39.6)	
Malpresentation	25 (14.8)	26 (15.4)	
Maternal request	13 (7.7)	15 (8.9)	
Failed induction of labor	13 (7.7)	7 (4.1)	
Macrosomia	11 (6.5)	12 (7. 1)	
Time to umbilical cord clamping, median (IQR)	65 (62,68)	65 (62,67)	0.188

IQR, interquartile range

### Primary outcome

Primary outcome data were available for 338 participants (169 in each group). There was no significant difference in neonatal hemoglobin between the two groups (Table 2). The mean neonatal hemoglobin at 48 to 96 h after birth was 194.3 ± 21.7 g/L in the intravenous infusion group and 195.2 ± 24.3 g/L in the intramyometrial group ( *p* = 0.715).

**Table 2:** Primary and secondary neonatal outcomes

Outcome	Intravenous group (*n* = 169)	Intramyometrial group (*n* = 169)	*P* value
Primary outcome			
Neonatal hemoglobin at 48–96 h, mean ± SD, g/L	194.3 ± 21.7 (*n* = 169)	195.2 ± 24.3 (*n* = 163)	0.715
Secondary neonatal outcomes			
Neonatal hematocrit at 48–96 h, mean ± SD, %	58.7 ± 6.7 (*n* = 169)	58.9 ± 7.8 (*n* = 163)	0.822
Gender, *n* (%)			0.828
Male	88 (52.1)	86 (50.9)	
Female	81 (47.9)	83 (49.1)	
Birth weight, mean ± SD, g	3414 ± 451.5	3452 ± 418.2	0.414
Birth weight > 4000 g, *n* (%)	22 (13.0)	20 (11.8)	0.742
Birth weight < 2500 g, *n* (%)	4 (2.4)	1 (0.6)	0.368
Apgar scores at 5 min, median (IQR)	10 (10, 10)	10 (10, 10)	1.000
Hypothermia, *n* (%)	5 (2.8)	4 (2.2)	1.000
Transcutaneous bilirubin, median (IQR), mg/dL			
Day 1	4.8 (3.9,5.6)	4.7 (3.8, 5.8)	0.694
Day 2	8.3 (7.0, 9.2)	8.2 (7.0, 9.7)	0.796
Day 3	10.7 (9.3, 11.9)	10.8 (9.3, 12.1)	0.745
Phototherapy for jaundice, *n* (%)	37 (21.9)	35 (20.7)	0.790
Neonatal anemia (hemoglobin < 145 g/L), *n* (%)	1 (0.6) (*n* = 169)	4 (2.5) (*n* = 163)	0.346
Neonatal infections, *n* (%)	3 (1.8)	6 (3.6)	0.499
Neonatal hypoglycemia	3 (1.8)	1(0.6)	0.615
NICU admission, *n* (%)	1 (0.6)	5 (3.0)	0.217
Neonatal death, *n* (%)	0 (0)	0 (0)	–
Feeding at 1 month, *n* (%)			0.645
Breast	90 (53.3)	86 (50.9)	
Bottle	21 (12.4)	27 (16.0)	
Mixed	58 (34.3)	56 (33.1)	
Re-admission within 30 days, *n* (%)	5 (3.0)	2 (1.2)	0.445

*NICU* neonatal intensive care unit

### Secondary neonatal outcomes

The neonatal hematocrit at 48 to 96 h after birth in the two groups were comparable either (58.7 ± 6.7% vs. 58.9 ± 7.8%, *p* = 0.822) (Table 2). The neonates in two groups were similar with respect to gender and birth weight. There were no obvious differences in transcutaneous bilirubin levels between the groups on Days 1, 2, and 3. Adverse neonatal outcomes, including NICU admission, phototherapy for jaundice and re-admission within 30 days were similar between the groups. The breastfeeding rate at 1 month was 53.3% in the intravenous group and 50.9% in the intramyometrial group (*p* = 0.645). No perinatal deaths occurred throughout the study period. Six neonates were admitted to the NICU. One patient in the intravenous group had respiratory distress syndrome. There are five infants in the intramyometrial group—two for neonatal infections, one for neonatal pneumonia, one for fetal ventricular septal defect and one for neonatal hemolytic jaundice. Although the NICU admission rate in the intramyometrial group (3.0%) was notably greater than that in the intravenous group (0.6%), this difference did not reach statistical significance (*p* = 0.217). The neonatal anemia rates did not differ significantly between the two groups (*p* = 0.346).

### Secondary maternal outcomes

There were no statistically significant differences seen in any measures of maternal blood loss and side-effects of oxytocin (Table 3). Although the intraoperative intravenous fluid volume in the intravenous group is 200 mL more than that in the intramyometrial group, there was no incidences of pulmonary edema or heart failure in either group. Hemoglobin was measured within 1 week before surgery and 2–3 days after surgery. The postpartum hemoglobin was 116.0 ± 11.8 g/L in the intravenous group and 117.4 ± 11.7 g/L in the intramyometrial group (*P* = 0.304). The peripartum change of hemoglobin showed no evident differences. No patient required a blood transfusion or ICU admission. The study observed a single case re-admission within 42 days due to acute appendicitis. The other characteristics of the cesarean operation and secondary maternal outcomes were shown in Table S1, S2. Per-protocol analysis had similar results to intent-to-treat analysis (Tables S3–S6).

**Table 3:** Secondary maternal outcomes

Outcomes	Intravenous group (*n* = 169)	Intramyometrial group (*n* = 169)	*P* value
Blood loss during cesarean, median (IQR), mL	320 (300, 400)	300 (300, 400)	0.524
Estimated blood loss within 24 h after delivery, median (IQR), mL	440 (380, 545)	421 (370, 545)	0.930
Estimated blood loss > 1000 mL, *n* (%)	1 (0.6)	3 (1.8)	0.615
Additional uterotonics for excessive bleeding, *n* (%)	24 (14.2)	30 (17.8)	0.373
Manual placental delivery	10 (5.9)	16 (9.5)	0.221
Side effects of oxytocin			
Hypotension, *n* (%)	23 (13.6)	26 (15.4)	0.646^a^
Tachycardia, *n* (%)	22 (13.0)	33 (19.5)	0.107^a^
Intraoperative intravenous fluid volume, median (IQR), mL	1000 (800,1200)	800 (700,1000)	< 0.001
Postpartum hemoglobin, mean ± SD, g/L	116.0 ± 11.8	117.4 ± 11.7	0.304
Peripartum hematocrit change (percentage points), median (IQR)	1.4 (0.3,3.4)	2.0 (0.3,4.0)	0.275
Blood transfusion, *n* (%)	0 (0)	0 (0)	–
ICU admission, *n* (%)	0 (0)	0 (0)	–
Re-admission within 42 days, *n* (%)	1 (0.6)	0 (0)	1.000^a^

^a^ Calculated with the *Fisher’s* exact test

## Discussion

In this randomized clinical trial, we found no statistically significant difference in the effect of intramyometrial and intravenous administration of oxytocin on neonatal hemoglobin among infants undergoing DCC during cesarean section. Mean hemoglobin of all the newborns was 194 g/L at 48 to 96 h after birth, which is consistent with other studies in Asia population [28–30]. This brief delay in umbilical cord clamping is known to increase the iron stores of the young infant by over 50% among babies born at both preterm and full-term [8, 31–34]. DCC does not pose a higher risk to mothers, which is also confirmed by our results. The guidelines do not provide a priority recommendation regarding the administration of oxytocin, whether through intramyometrial or intravenous route [18]. The administration of oxytocin through intramyometrial injection is frequently observed in clinical practice as a common use. In Japan, injection of oxytocin intramyometrially is performed in over half of all cesarean sections, either alone or in combination with the intravenous route [17]. Although several studies have attempted to identify an appropriate administration route of oxytocin that ensures acceptable uterine contraction and minimal hemodynamic side-effects [35–37], their conclusions were not entirely consistent. Our study did not find any obvious difference in uterine contraction between these two routes of oxytocin administration in the initial minute after delivery. The choice of oxytocin administration during a cesarean section mostly depending on the experience and preference of the anesthesiologist or obstetrician.

Some clinicians might be worried about the increased intraoperative fluid loading [38]. Our study showed that the additional 200 mL of fluid volume in the intravenous group seemed to have no ability to cause acute cardiopulmonary complications, as no patients experienced pulmonary edema or heart failure during the study. Notably, when comes to patients with pre-eclampsia who are at high risk of pulmonary edema, we suggest that the surgeon’s expertise and the specific conditions of parturients should be taken into consideration when selecting an appropriate route for oxytocin administration.

Although current clinical practice guidelines recommend DCC as the standard management of the third stage of labor, the coverage of this intervention was limited [39]. A nationwide survey of 126 hospitals in China revealed that the implementation rate of DCC was 41.6% [40], and the rate is merely 33% in cesarean sections. The implementation of DCC in China is currently hindered by organizational challenges and pragmatic difficulties in clinical practice. The majority of hospitals in China lack standardized guidelines in DCC practice. ECC remains the preferred practice for cesarean sections. Our research has a positive significance in enhancing the awareness of umbilical cord management, standardizing the operational procedures of DCC, and facilitating its wider implementation.

The strengths of this work are as follows: it is the first randomized trial, to our knowledge, that evaluates the effects of different administration routes of oxytocin in neonates with DCC specifically in elective cesarean delivery at term. Another strength of this study is that it is a multicenter study, conducted strictly following the protocol.

There were some limitations in our study. First, neonatal hemoglobin data for 22 patients were not available. The whole sample size is 4 less than that calculated. Based on the existing results, it can be speculated that even if 4 sample was added to the analysis, it is unlikely that they would cause significant differences in the results. Second, we implemented DCC for a duration of one minute, leaving it uncertain whether extended delayed umbilical cord clamping of 2 or 3 min would result in additional adverse effects. Third, the outcomes of this study were only the short-term effects; however, the main advantage of DCC in a term infant is the higher iron storage at 6 months of age. A long-term study is still required to ascertain the effect of DCC on infants aged 6 months or older.

## Conclusion

In conclusion, this study showed that the route of oxytocin administration during elective cesarean delivery does not significantly impact its effectiveness in women undergoing DCC. Although the intramyometrial injection of oxytocin is not recommended by most guidelines, it remains a common practice in some countries. The choice of oxytocin administration during a cesarean section depend mainly on the experience and preference of the anesthesiologist or obstetrician. The results of our study may have relevant impact on future clinical practice and guidelines.

### Electronic Supplementary Material

Below is the link to the electronic supplementary material.

Supplementary material 1 (PDF 214 kb)

## Data Availability

Data can be obtained from the corresponding author upon reasonable request.
