# Supplementary material for: Effect of administration routes of oxytocin on hemoglobin in neonates with delayed umbilical cord clamping: a multi-centre randomized controlled clinical trial
Source: Arch Gynecol Obstet. 2024 May 16;310(2):991–9. doi: 10.1007/s00404-024-07543-w (PMC11258157; doi:10.1007/s00404-024-07543-w)
Supplement: Supplementary file 1 — Supplementary material 1 (PDF 214 kb) [file 404_2024_7543_MOESM1_ESM.pdf]

# Supplementary Tables

**( intent-to-treat analysis:S1-S2 )**

**Supplemetary Table S1** Characteristics of the cesarean operation

| Characteristics | Intravenous Group (n=169) | Intramyometrial Group (n=169) | P-value |
|---|---|---|---|
| Total surgical time[a], median (IQR), min | 45 (33, 55) | 47 (39, 55) | 0.193 |
| Mechanical or surgical interventions, n (%) | 4 (2.4) | 8 (4.7) | 0.240 |
| Compressive uterine sutures | 2 (1.2) | 3 (1.8) | |
| Vessel ligation | 1 (0.6) | 4 (2.4) | |
| Intrauterine tamponade balloon | 0 (0) | 0 (0) | |
| Uterine packing with gauze | 1 (0.6) | 1 (0.6) | |
| Skin incision type | | | 0.547 |
| Pfannenstiel | 157 (92.9) | 154 (91.1) | |
| Vertical | 12 (7.1) | 15 (8.9) | |
| Cesarean myomectomy | 4 (2.4) | 3 (1.8) | 1.000 |
| Tubal ligation | 6 (3.6) | 4 (2.4) | 0.521 |

**Supplementary Table S2** Secondary maternal outcomes

| | Intravenous Group (n=169) | Intramyometrial Group (n=169) | P-value |
|---|---|---|---|
| Blood loss within 2 hours after delivery, median (IQR), ml | 60 (50, 90) | 70 (50, 108) | 0.148 |
| Estimated blood loss > 2000 ml, n (%) | 0 (0) | 0 (0) | - |
| Calculated blood loss*, median (IQR), ml | 236 (43,551) | 308 (61,656) | 0.223 |
| Calculated blood loss > 1000 ml, n (%) | 12 (7.1) | 18 (10.7) | 0.251 |
| Calculated blood loss > 2000 ml, n (%) | 1 (0.6） | 1 (0.6) | 1.000 |
| Hemoglobin | | | |
| Peripartum change, median (IQR), g/L | 5.0 (0.5, 12.0) | 7.0 (-1.0, 13.0) | 0.793 |
| Peripartum decrease ≥ 20 g/L | 16 (9.5) | 12 (7.1) | 0.430 |
| Hematocrit | | | |
| Peripartum change (percentage points) | 1.4 (0.3,3.4) | 2.0 (0.3,4.0) | 0.275 |
| Peripartum decrease of >10 percentage points | 1 (0.6) | 2 (1.2) | 1.000 |
| Additional uterotonics for excessive bleeding, n (%) | 24 (14.2) | 30 (17.8) | 0.373 |
| Ergometrine | 7 (4.1) | 7 (4.1) | 1.000 |
| Misoprostol | 0 (0) | 1 (0.6) | 1.000[a] |
| Carboprost | 18 (10.7) | 22 (13.0) | 0.501 |

**( Per-protocol analysis set: S3- S6)**

Three patients who did not receive intervention as randomized were excluded from the per-protocol data set.

**Supplementary Table S3** Baseline Characteristics of the two groups

| Characteristics | Intravenous Group (n=168) | Intramyometrial Group (n=167) | P-value |
|---|---|---|---|
| Maternal age, mean± SD, years | 31.6 ± 4.7 | 31.4 ± 4.4 | 0.639 |
| Gestational age at delivery, median (IQR), week, | 39 (38.7,39.2) | 39 (38.6,39.3) | 0.493 |
| Primigravida, n (%) | 95 (56.5) | 89(53.3) | 0.559 |
| Pre-delivery BMI, median (IQR), kg/m$^2$ | 27.8 (25.7,30.0) | 28.0 (26.0,30.4) | 0.258 |
| Preoperative haemoglobin (g/L, mean ± SD) | 122.8 ± 10.6 | 123.7 ± 10.4 | 0.417 |
| hemoglobin < 110 g/L, n (%) | 15(8.9) | 11 (6.6) | 0.423 |
| 71-89 | 0 | 0 | |
| 90-99 | 2 (1.2) | 0 | 0.481 |
| 100-109 | 13(7.7) | 11(6.6) | 0.683 |
| Previous cesarean section, n (%) | | | 0.793 |
| 1 | 59 (35.1) | 64 (38.3) | |
| ≥2 | 4 (2.4) | 3 (1.8) | |
| Hypertension disorder, n (%) | 9 (5.4) | 10 (6.0) | 0.803 |
| Gestational diabetes, n (%) | 31 (18.5) | 23 (13.8) | 0.244 |
| Intrahepatic cholestasis of pregnancy, n (%) | 2 (1.2) | 1 (0.6) | 1.000 |
| Hepatitis B virus infection, n (%) | 3 (1.8) | 8 (4.8) | 0.123 |

**Supplementary Table S4** Characteristics of the cesarean operation

| | Intravenous Group (n=168) | Intramyometrial Group (n=167) | P-value |
|---|---|---|---|
| Total surgical time[a], median (IQR), min | 45 (33.2,55.0) | 47 (39.0,55.0) | 0.186 |
| Placental delivery, n(%) | | | 0.215 |
| Spontaneous | 158 (94.0) | 151 (90.4) | |
| Manual | 10 (6.0) | 16 (9.6) | |
| Blood loss during cesarean, median (IQR), ml | 320 (300,400) | 300 (300,400) | 0.524 |
| Blood loss within 2 hours after delivery, median (IQR), ml | 60 (50， 90) | 70 (50,107) | 0.182 |

| | | | |
|---|---|---|---|
| Mechanical or surgical interventions, n (%) | 4 (2.4) | 8 (4.8) | 0.235 |
| Compressive uterine sutures | 2 (1.2) | 3 (1.8) | |
| Vessel ligation | 1 (0.6) | 4 (2.4) | |
| Intrauterine tamponade balloon | 0 (0) | 0 (0) | |
| Uterine packing with gauze | 1 (0.6) | 1 (0.6) | |
| Side effects of oxytocin | | | |
| Hypotension, n (%) | 23 (13.7) | 26 (15.6) | 0.591* |
| Tachycardia, n (%) | 23 (13.1) | 33 (19.8) | 0.091* |
| Intraoperative intravenous fluid volume, median (IQR), ml | 1000 (800,1200) | 800 (700,1000) | < 0.001 |
| Skin incision type | | | 0.536 |
| Pfannenstiel | 156 (92.9) | 152 (91.0) | |
| Vertical | 12 (7.1) | 15 (9.0) | |
| Cesarean myomectomy | 4 (2.4) | 2 (1.2) | 0.686 |
| Tubal ligation | 6 (3.6) | 4 (2.4) | 0.755 |

*Calculated with the *Fisher* exact test.

**Supplementary Table S5** Primary and secondary neonatal outcomes

| | Intravenous Group (n=168) | Intramyometrial Group (n=167) | P value |
|---|---|---|---|
| Primary Outcome | | | |
| Neonatal hemoglobin at 48-96 h,mean ± SD, g/L | 194.5 ± 21.6 (n =168) | 195.4 ± 24.3 (n =162) | 0.727 |
| Secondary Neonatal Outcomes | | | |
| Neonatal hematocrit at 48-96 h, mean ± SD, % | 58.8 ± 6.7 (n =168) | 58.9 ± 7.7 (n =162) | 0.832 |
| Gender, n (%) | | | 0.786 |
| Male | 88 (52.4) | 85 (50.9) | |
| Female | 80 (47.6) | 82 (49.1) | |
| Birth weight, mean ± SD, g | 3420.4 ± 445.3 | 3454.9± 418.1 | 0.465 |
| Birth weight >4000 g, n (%) | 22 (13.1) | 20 (12.0) | 0.757 |
| Low birth weight, n (%) | 3 (1.8) | 1 (0.6) | 0.619 |
| Apgar scores at 5 minutes, median (IQR) | 10 (10,10) | 10 (10,10) | 1.000 |
| Hypothermia, n (%) | 4 (2.4) | 4 (2.4) | 1.000 |
| Transcutaneous bilirubin, median (IQR), mg/dL | | | |
| Day 1 | 4.8 (3.9,5.6) | 4.7 (3.8,5.8) | 0.788 |
| Day 2 | 8.4 (7.0,9.2) | 8.2 (7.0,9.6) | 0.910 |
| Day 3 | 10.7 (9.3,12.0) | 10.8 (9.3,12.0) | 0.897 |
| Phototherapy for jaundice, n (%) | 37 (22.0) | 33 (19.8) | 0.610 |

| | | | |
|---|---|---|---|
| Neonatal anemia (hemoglobin <145 g/L), n (%) | 1 (0.6)<br>(n =168) | 4 (2.5)<br>(n =162) | 0.346 |
| Neonatal infections, n (%) | 3 (1.8) | 6 (3.6) | 0.493 |
| Neonatal hypoglycemia | 3 (1.8) | 1 (0.6) | 0.619 |
| NICU admission, n (%) | 1 (0.6) | 5 (3.0) | 0.214 |
| Neonatal death, n (%) | 0 (0) | 0 (0) | - |
| Feeding at one month, n (%) | | | 0.625 |
| Breast | 89 (53.0) | 86 (51.5) | |
| Bottle | 21 (12.5) | 27 (16.2) | |
| Mixed | 58(34.5) | 54 (32.3) | |
| Readmission within 30 days, n (%) | 5 (3.0) | 2 (1.2) | 0.450 |

a,Calculated with the *Fisher's* exact test.

**Supplementary Table S6** Secondary maternal outcomes

| | Intravenous Group (n=168) | Intramyometrial Group (n=167) | P value |
|---|---|---|---|
| Estimated blood loss within 24 hours after delivery, median (IQR), ml | 439(380,542) | 420.5(370,540) | 0.907 |
| Estimated blood loss > 1000 ml[b], n (%) | 1 (0.6) | 3 (1.8) | 0.611 |
| Estimated blood loss > 2000 ml, n (%) | 0 (0) | 0 (0) | - |
| Calculated blood loss*, median (IQR), ml | 237 (42,554) | 304 (57,653) | 0.293 |
| Calculated blood loss > 1000 ml, n (%) | 12 (7.1) | 17 (10.2) | 0.323 |
| Calculated blood loss > 2000 ml, n (%) | 1 (0.6) | 0 (0) | 1.000[a] |
| Hemoglobin | | | |
| Postpartum, mean ± SD, g/L | 116.1±11.8 | 117.3±11.7 | 0.356 |
| Peripartum change, median (IQR), g/L | 5.0 (0.2,12.0) | 7.0 (-1.0,13.0) | 0.827 |
| Peripartum decrease ≥ 20 g/L | 16 (9.5) | 12 (7.2) | 0.439 |
| Hematocrit | | | |
| Peripartum change (percentage points) | 1.4 (0.3,3.4) | 1.9 (0.3,3.9) | 0.352 |
| Peripartum decrease of >10 percentage points | 1(0.6) | 1(0.6) | 1.000 |
| Additional uterotonics for excessive bleeding, n (%) | 24 (14.3) | 30 (18.0) | 0.360 |
| Ergometrine | 7 (4.2) | 7 (4.2) | 0.991 |
| Misoprostol | 0 (0) | 1 (0.6) | 0.499[a] |
| Carboprost | 18 (10.7) | 22 (13.2) | 0.488 |
| ICU admission, n (%) | 0 (0) | 0 (0) | |
| Blood transfusion, n (%) | 0 (0) | 0 (0) | |
| Readmission within 42 days, n (%) | 1 (0.6) | 0 (0) | 1.000[a] |

a,Calculated with the Fisher's exact test. b, Postpartum hemorrhage was defined as a estimated blood loss > 1000 ml.

*Calculated blood loss = estimated blood volume $\times$ (preoperative hematocrit – postoperative hematocrit) / preoperative hematocrit (where estimated blood volume [mL]=weight [kg] $\times$85).
